# Population Genomics of *Bettongia lesueur*: Admixing Increases Genetic Diversity with no Evidence of Outbreeding Depression

**DOI:** 10.3390/genes10110851

**Published:** 2019-10-28

**Authors:** Kate Rick, Kym Ottewell, Cheryl Lohr, Rujiporn Thavornkanlapachai, Margaret Byrne, W. Jason Kennington

**Affiliations:** 1 Biodiversity and Conservation Science, Department of Biodiversity, Conservation and Attractions, Locked Bag 104, Bentley Delivery Centre, Crawley, WA 6983, Australia; cheryl.lohr@dbca.wa.gov.au (C.L.); rujiporn.sun@dbca.wa.gov.au (R.T.); margaret.byrne@dbca.wa.gov.au (M.B.); 2 School of Biological Sciences, The University of Western Australia, Crawley, WA 6009, Australia; jason.kennington@uwa.edu.au

**Keywords:** genetic diversity, translocation, admixing, outbreeding depression, *Bettongia lesueur*, fitness, genomics, SNP, conservation

## Abstract

Small and isolated populations are subject to the loss of genetic variation as a consequence of inbreeding and genetic drift, which in turn, can affect the fitness and long-term viability of populations. Translocations can be used as an effective conservation tool to combat this loss of genetic diversity through establishing new populations of threatened species, and to increase total population size. Releasing animals from multiple genetically diverged sources is one method to optimize genetic diversity in translocated populations. However, admixture as a conservation tool is rarely utilized due to the risks of outbreeding depression. Using high-resolution genomic markers through double-digest restriction site-associated sequencing (ddRAD-seq) and life history data collected over nine years of monitoring, this study investigates the genetic and fitness consequences of admixing two genetically-distinct subspecies of *Bettongia lesueur* in a conservation translocation. Using single nucleotide polymorphisms (SNPs) identified from 215 individuals from multiple generations, we found an almost 2-fold increase in genetic diversity in the admixed translocation population compared to the founder populations, and this was maintained over time. Furthermore, hybrid class did not significantly impact on survivorship or the recruitment rate and therefore we found no indication of outbreeding depression. This study demonstrates the beneficial application of mixing multiple source populations in the conservation of threatened species for minimizing inbreeding and enhancing adaptive potential and overall fitness.

## 1. Introduction

Mammal species throughout the world have been subject to significant threats, particularly from invasive species [1,2], and frequently persist in small populations. Inbreeding, the crossing between closely related individuals, is often exacerbated in these small and isolated populations and can lead to increased expression of deleterious recessive alleles, which probably contributes most strongly to inbreeding depression and a subsequent reduction in fitness [3,4,5,6]. In small fragmented populations, random genetic drift can also result in the loss of heterozygosity and reduces allelic richness [7,8]. This loss of genetic variation leads to a reduction in evolutionary potential, thereby increasing vulnerability of populations to environmental (e.g. environmental stress as a result of drought) and demographic stochasticity (i.e., fluctuations in local survivorship and fecundity) [3,9]. Subsequently, small and vulnerable populations can be diminished through a phenomenon known as the ‘extinction vortex’, where a positive feedback loop between the size of the population and the average fitness of its members, renders populations at an increased risk of extirpation [10,11]. 

Translocations and reintroductions are widely proposed and used in conservation to counteract these demographic and genetic threats to small fragmented populations [8,12,13,14,15]. Translocations and reintroductions are often used to establish or re-establish new populations of threatened species but can also be used as a means to supplement or augment existing populations to restore genetic diversity to depauperate populations. Management of genetic diversity is important to consider at all stages of the translocation process, including within the founding population and in ongoing management [16,17]. In particular, it is becoming more common for translocations and reintroduction sites to be established where threatening processes can be effectively managed, such as predator free islands or fenced enclosures, resulting in the isolation of these populations from extant populations [8,18]. These physically isolated populations lack gene flow and therefore require careful planning and ongoing genetic monitoring, including possible future supplementation, to prevent further loss of diversity, especially if population sizes are consistently small. 

Genetic variation is one factor which may influence the success of a translocation or reintroduction [17,19]. In planning for translocations, the loss of genetic variation can be minimized by increasing the number of founders or by admixing animals from different source populations [9,19], although the relative effectiveness of the two is yet to be fully understood [19]. Reintroduced populations for threatened species are often established from a small number of founder individuals as a result of rarity of wild populations and the high cost associated with translocation and captive breeding programs [8]. Thus, as proposed by Biebach and Keller [19], releasing animals from different sources might be more effective than releasing many animals from a single source to optimize genetic diversity in translocated populations. 

Further, the introduction of individuals into inbred populations can provide a ‘genetic rescue’ effect by infusing new genetic variation and relieving deleterious effects of inbreeding leading to improved fitness [20]. This phenomenon is an example where admixture can be used to improve conservation outcomes for threatened species and has been recorded in a range of mammals including; the mountain pygmy possum (*Burramys parvus*) [21], the Florida panther (*Puma concolor coryi*) [7,22], the Mexican wolf (*Canis lupus baileyi*) [7], the Scandinavian wolf (*Canis lupus*) [23], the Scandinavian arctic fox (*Vulpes lagopus*) [24], and the Rocky Mountain bighorn sheep (*Ovis Canadensis*) [25]. Genetic rescue can occur through an increase in fitness attributed to increased heterozygosity, known as heterosis [20], or by adaptive evolution, where phenotypes favored by natural selection are produced through introducing or combining new genotypes achieved by outcrossing [26]. In admixed populations, genetic rescue in the F_1_ generation would be expected due to differences in allele frequencies between parental types, leading to high heterozygosity and subsequent high fitness [20,26].

A possible unintended consequence of using admixture for conservation purposes is outbreeding depression, the reduction in fitness following the crossing of genetically divergent populations, which may be due to intrinsic factors, where co-adapted gene complexes may be disrupted leading to genic incompatibilities of hybridized populations, or extrinsic factors, where introduced alleles dilute locally adaptive alleles leading to reduced adaptation to the local environmental conditions [27,28]. As this genetic breakdown requires recombination, outbreeding depression may only be detected after several generations of outcrossing (F_2_ and beyond) [27]. Few studies, especially for vertebrates, monitor populations long-term nor extend beyond the F_2_ generation and, therefore, the frequency and severity of outbreeding depression in wild populations is yet to be fully understood [26,29]. Despite this, outbreeding depression has still been observed in a wide range of animal taxa including copepods [30], insects [31], birds [32], fish [33,34], and mammals [35].

Admixture as a conservation method can be controversial due to concerns regarding whether the crossing of distinct populations will have the desired effect: to counteract inbreeding depression and recover genetic diversity; or the opposite effect: outbreeding depression and lack of viability [26,27,28]. Caution is particularly warranted if the populations outcrossed are between different taxa, are adapted to different environmental conditions, have been separated for long periods of time, or a combination of these circumstances [36]. As a result, relatively few cases of admixture have been implemented despite its potential benefits to small and declining populations, with only approximately 20 cases of genetic rescue being documented on wild populations [37]. 

**Figure 1:**
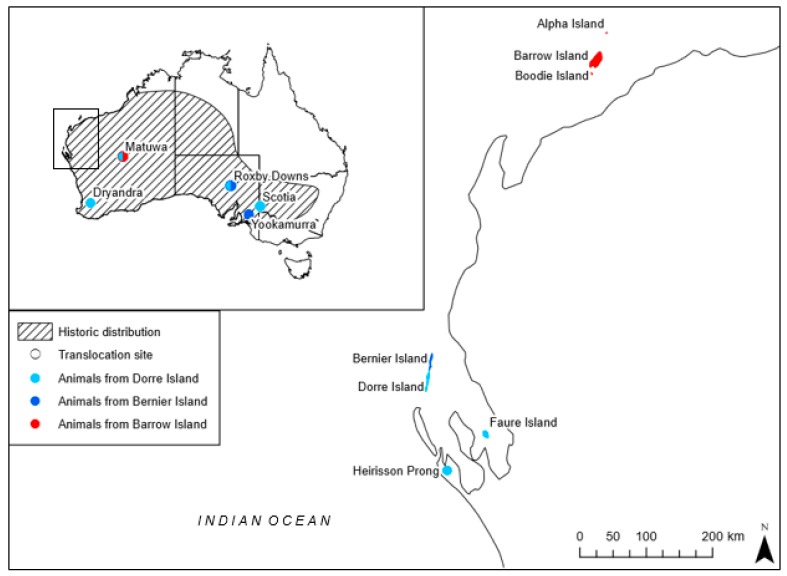
Location of natural and translocated populations of *Bettongia lesueur* within its historic distribution (adapted from Short & Turner [38]). Source population origin for each translocation is indicated where ● represents individuals from Dorre Island (Shark Bay subspecies *B. l. lesueur*), ● represents individuals from Bernier Island (Shark Bay subspecies *B. l. lesueur*) and ● represents individuals from Barrow Island (Barrow Island subspecies *B. lesueur* unnamed subspecies). Translocated populations with admixing show two colors; Roxby Downs admixed from the two Shark Bay populations and Matuwa admixed from the two subspecies.

In this study, we take advantage of a reintroduction of the threatened burrowing bettong (*Bettongia lesueur*) to investigate the genetic and demographic implications of admixing highly diverged source populations in a translocation. *Bettongia lesueur* is listed as Vulnerable under the Australian Environmental Protection and Biodiversity Conservation Act 1999, and Conservation Dependent under the Western Australian Biodiversity Conservation Act 2016. Once widespread across two-thirds of mainland Australia (Figure 1), *B. lesueur* currently occupies less than 0.01% of its historical range, persisting in three naturally occurring populations in Western Australia; Bernier and Dorre Island in Shark Bay, and Barrow Island, off the Pilbara coast [38,39,40]. Individuals from the Shark Bay population are significantly larger and genetically distinct from their Barrow Island relatives, and subsequently, according to the key proposed by Wiens and Penkrot [41], are deemed separate subspecies; *Bettongia lesueur lesueur* (Shark Bay) and *Bettongia lesueur* unnamed subspecies (Barrow Island). Multiple reintroductions to mainland Australia have been attempted (Figure 1), but populations have only established when transferred into predator-free enclosures [40] as the species is particularly vulnerable to introduced mammalian predators [42,43,44]. The translocation to Matuwa (26°13′S, 121°33′E) is the only reintroduction involving *B. lesueur* to use both subspecies. 

The long-term geographical isolation (over 8000 years) and genetic distinctness [45] between the *B. lesueur* subspecies raises concerns regarding whether the admixing of these source populations will result in a single randomly mating population with no deleterious effects on fitness. According to the decision tree for outbreeding depression published by Frankham et al. [28], the risk of outbreeding depression in this translocation population is high due to the absence of gene flow between the two subspecies in the last 500 years. A previous study on the early state of this translocation using microsatellite markers and mitochondrial DNA found higher levels of genetic diversity in the mixed Matuwa population relative to one founder population (Shark Bay), but not the other (Barrow Island) [45]. They also detected asymmetrical introgression between the two sub-species due to higher than expected crosses between smaller-sized Barrow Island males and larger-sized Shark Bay females [45].

Here, we use next-generation sequencing using double-digest restriction site-associated DNA sequencing (ddRAD-seq), in conjunction with accompanying long term fitness data from multiple generations of outcrossing to; (i) investigate the difference in genetic diversity between the founder populations and the admixed translocated population at nine years post-release, (ii) determine whether progressive introgression has led to the breakdown of genetic differentiation between the two populations over time, and (iii) examine whether survivorship and recruitment rate differ across hybrid generations, thereby testing for outbreeding depression. 

## 2. Materials and Methods 

### 2.1. Translocation History

In 2012, there were eight successful reintroductions of *B. lesueur* which were considered wild, self-sustaining, and have survived and reproduced for more than five years [46]. These include reintroductions to Heirisson Prong in 1992, Boodie Island in 1993, Roxby Downs (Arid Recovery) in 1999–2000, Faure Island in 2002, Scotia in 2004, Yookamurra in 2007, Alpha Island in the Montebello Archipelago in 2010, and Matuwa in Western Australia also in 2010 (Figure 1). However, the population at Heirisson Prong is no longer extant. 

The Matuwa population was established using individuals from Shark Bay, which were sourced from the Return to Dryandra Field Breeding Facility (32°48′S, 117°0′E), hereafter referred to as Dryandra (DRY), and individuals directly sourced from Barrow Island (BWI) (20°51′S, 115°24′E). Stock individuals for Dryandra were originally sourced from Dorre Island, Shark Bay (Figure 1). All Matuwa founders were released incrementally between January and October in 2010 (Table 1). 22 of the Shark Bay individuals released in August were recaptured and moved outside the translocation pen two months after initial release. This population at Matuwa has been successfully established, with the population increasing from the initial 176 individuals in 2010 to over 900 individuals in 2018 [47].

### 2.2. Data Collection and Sample Selection

All genetic samples, including founder individuals, were collected by the Department of Biodiversity, Conservation and Attractions (DBCA) during regular population monitoring (capture-mark-recapture) trapping sessions at Matuwa between 2010 and 2018. Each trapped individual was microchipped, had a 2 mm tissue sample taken from its ear (stored in 70% ethanol), weighed, measured (head and pes length), and had their age and sex recorded. The reproductive status of females was also recorded by determining whether pouch young were present (including number and approximate length) or if they showed signs of lactation. Individuals were classified as adults if they had pouch young, one or more teats showing signs of lactation, or had a fully developed pouch or testes. 

Data from individual trapping histories were used to assess fitness proxies (below). An additional year of trapping data (2019) was included in the fitness analyses but excluded from the genetic analyses as it was not available prior to the sample selection process. The presence of pouch young was not used in fitness analyses as trapping generally occurs around May when *B. lesueur* is less likely to produce young. 

A stratified sampling design was used to select individuals for analysis in order to maximize variation in survivorship and year of collection. Using the capture-mark-recapture dataset, individuals were stratified based on the number of years of trapping history; four or more years of capture histories were classified as ‘long lived’ and less than four years of capture histories were classified as ‘short lived’. This stratification is based on the expected longevity of *B. lesueur* being more than three years [41]. For the purpose of this study it is assumed that individuals not captured again are deceased. Furthermore, due to the age of individuals being unknown at first capture, the age of an individual was not considered when classifying individuals as ‘long lived’ or ‘short lived’. Founder individuals, representing each of the source population’s ancestry were randomly selected within the ‘long lived’ strata category (Barrow Island *N* = 12, Shark Bay *N* = 12) or ‘short lived’ strata category (Shark Bay *N* = 12, Barrow Island *N* = 12). Progeny samples (i.e., all non-founding individuals) were further stratified based on when the individual was first trapped; individuals first trapped between 2010 and 2012 were classified as ‘caught early’ and individuals first trapped between 2010 and 2013 were classified as ‘caught late’. Progeny samples (*N* = 174) were then randomly selected within each of these strata categories; ‘long lived’, ‘short lived’, ‘caught early’ and ‘caught late’ (Table S1). Years 2016, 2017, and 2018 had no ‘long lived’ individuals due to the lack of more than four years of trapping data and as such were randomly selected.

### 2.3. DNA Extraction and ddRAD-seq Library Preparation

Genomic DNA was extracted from the biopsied ear tissue by the ‘salting-out’ method [48] with a modification of 10 mg/mL Proteinase K being added to 300 µL TNES and incubated at 56 ℃ [45]. Extracts were quantified using Qubit Fluorometric Quantitation as per manufacturer’s instructions. 

Double-digest restriction site-associated DNA sequencing (ddRAD-seq) libraries were prepared in batches of 96 samples (including a negative control and a minimum of two replicates within a plate and between plates). A total of 200 ng of DNA for each of 228 samples was sent to the Australian Genome Research Facility (AGRF) for ddRAD library preparation and Illumina sequencing. In brief, 50 ng of gDNA for each sample was digested using two restriction enzymes (PstI and Mspl) and ligated with unique barcoded adapters compatible with the restriction site overhang. Samples were purified and size selection (280–375 bp) carried out on the Blue Pippin (Sage Science). Libraries were PCR amplified with indexed primers and sequencing carried out on the Illumina NextSeq 500 with 150 cycles in HIGH-output mode over two flowcells. Short sequencing reads can be accessed via the Bioplatforms Australia data portal for the Oz Mammals Genomics Initiative (https://data.bioplatforms.com/organization/about/bpa-omg). 

### 2.4. Sequence Processessing, Quality Control, and SNP Filtering 

STACKS v1.35 pipeline [49] was used to process the raw sequencing reads and assemble reads de novo to identify biallelic single nucleotide polymorphisms (SNPs). Through the *process_radtags* module, reads were cleaned and demultiplexed, truncated to 120bp and filtered for overall quality with samples with fewer than 400,000 reads (*N* = 10) excluded from further analysis. Using the *ustacks* module, RAD loci were identified. Optimal parameters were systematically selected to maximize the number of polymorphic loci using the r80 optimization approach described by Paris et al. [50] while also minimizing errors calculated between samples replicated within plates as recommended by Mastretta-Yanes et al. [51]. Optimization tests were run testing the value of *m* (the minimum number of identical raw reads required to create a stack) from three to seven, and *M* (the minimum number of mismatches allowed between loci when processing a single individual) from one to eight. The effects of *m* and *M* parameter selection were evaluated based on the number of assembled loci, the number of polymorphic loci, and the number of SNPs for each value. The effects of *m* on stack depth/coverage was also evaluated. The optimal value of both *m* and *M* was chosen based on a trade-off between the highest retrieval of SNPs while minimizing errors. Following Paris et al. [50] recommendation, n (the minimum number of mismatches allowed between loci when building the catalogue) was set to equal *M*. Using the *ustacks* output, the *cstacks* module constructed a catalogue of consensus loci among individuals and the *population* module then restricted the data analysis to only the first SNP per locus minimizing linked SNPs for downstream analysis and required at least 20% of individuals in the founder population or admixed translocated population to process a locus for that population (*r* = 0.2). The resulting SNP dataset was output in a VCF format (http://dx.doi.org/10.17632/vg983472v3.1).

To further improve genotyping quality for this study, loci with a minor allele frequency (MAF) less than 5%, minimum heterozygosity less than 5%, and a maximum heterozygosity greater than 95% were excluded using the program TASSEL Version 5 [52]. Two data sets were then generated, one including loci that were genotyped in more than 80% of individuals (80% complete) and the second including loci that were genotyped in more than 95% of individuals (95% complete). The first dataset maximizes the number of SNPs called and is used to assess genetic diversity, while the second dataset gives greater power and accuracy to determine ancestry. PLINK [53] was then used to further filter both datasets removing loci that were in linkage disequilibrium (LD) and did not conform to Hardy-Weinberg expectations following a Bonferroni correction. LD was measured based on the squared correlation coefficient (r^2^) between alleles in each individual. Any loci with an r^2^ value greater than 0.2 was considered in LD and excluded. To avoid removing loci that are in LD or depart from Hardy-Weinberg expectations due to population structure, LD and Hardy-Weinberg were filtered separately for each founder population and for each year for the translocated population.

### 2.5. Genetic diversity

Analyses of genetic diversity were based on the 80% complete dataset, with samples grouped according to their population of origin (Founders: BWI *N* = 22, DRY *N* = 24) or years of collection (Matuwa: 2010 *N* = 4, 2011 *N* = 15, 2012 *N* = 19, 2013 *N* = 37, 2014 *N* = 33, 2015 *N* = 16, 2016 *N* = 15, 2017 *N* = 15, 2018 *N* = 15). To evaluate the genetic diversity within each of these groups, standard diversity indices were calculated in R version 3.5.1 using the package *hierfstat* [54], including; average expected heterozygosity (H*_e_*) and average observed heterozygosity (H*_o_*), number of alleles rarefied by sample size or allelic richness (A*_R_*), and the inbreeding coefficient (F*_IS_*) to quantify a deficit or excess in heterozygotes relative to random mating. The significant deviation of F*_IS_* values from Hardy-Weinberg Equilibrium were determined by calculating bootstrap confidence intervals corrected for multiple comparisons for 10,000 iterations. Individuals from 2010 were excluded from this analysis due to a small sample size (*N* = 4). For each genetic diversity or inbreeding parameter, differences among collection years and founder populations were tested using a Friedman’s ANOVA, with samples paired by locus using R version 3.5.1 [55]. To assess individual genetic diversity, individual multi-locus heterozygosity (MLH), which measured the proportion of heterozygous loci within an individual was calculated using the R package *inbreedR* [56]. MLH was calculated to determine whether there was a difference in heterozygosity between sexes in hybrid and non-hybrid individuals using a Wilcoxon’s signed-rank tests.

### 2.6. Population Genetic Structure

Genetic distance between founder populations and translocated populations was determined using pairwise *F*_ST_ in Arlequin Version 3.5 [57] using the underlying pairwise distance matrix and 10,000 permutations. Significance values were corrected for multiple tests using the Bonferroni correction. Although the collection year in 2010 had a small sample size, Nazareno et al. [58] demonstrates that a minimum of two samples with a large number of SNPs (>1500) was still suitable to accurately estimate *F*_ST_. It was, therefore, included in this analysis. 

Due to the social structuring associated with warren use of *B. lesueur* [59] in conjunction with the limited dispersal range within the fenced enclosure, related individuals were expected within this population. Therefore, a Discriminant Analysis of Principal Components (DAPC) was used as a multivariate method to identify and describe clusters of related individuals [60] and visualize the extent of genetic mixing and differentiation between the founder populations and translocated population over time using the R package *adegenet* [61]. DAPC groups individuals to achieve the largest between-group variance and the smallest within-group variance using linear combinations of alleles [60]. To achieve this, it performs Principal Component Analysis as a prior step to the Discriminant Analysis. The *find.cluster* command was run with the number of component (PCs) set to allow 90% of cumulative variance to be retained (100–110 PCs) with two clusters. 

The program fastSTRUCTURE [62] was used to assess the extent of genetic mixing in the translocated population and any changes in genetic composition over time. This program is similar to STRUCTURE [63] in estimating the proportion of each individual’s genome that originated from a number of gene pools (K) but is based on a variational Bayesian framework for posterior inference enabling the extrapolation of population structure from large SNP datasets [62]. Furthermore, it uses heuristic scores to identify the number of source populations represented in the dataset, where the likelihood of different K values (1–5) were compared. The variational Bayesian technique approximates the log marginal likelihood of the data by proposing a group of tractable parametric posterior distributions (i.e., variational distributions) and finds the optimal member of this group to best approximate the marginal likelihood of the data (*K* = 2). To ensure consistency in approximated ancestry proportions in our dataset, we also calculated Bayesian Markov chain Monte Carlo (MCMC) hybrid indexes using the R package gghybrid [64]. This package utilizes information from biallelic genomic data to quantify the genetic contribution of hybridizing species to individuals of unknown ancestry through scoring genotypes according to the parent-of-origin of each allele [64,65]. 

To further assess patterns of introgression and changes in genetic composition within the admixed Matuwa population, variation in allele frequencies were calculated over time. This was achieved through VCFtools [66] where alleles fixed for either founder population were calculated by filtering both founder populations for loci with an *F*_ST_ value of one. Allele frequencies for each of these loci (*N* = 954) were then calculated for each year of the Matuwa population in GenAlEx version 6.5 [67,68].

### 2.7. Testing for Outbreeding Depression

Using the 95% complete dataset, individuals from the Matuwa translocated population were assigned to one of six hybrid classes using the program NEWHYBRIDS [69]. Due to the program being unable to handle large genomic datasets, the 300 most informative loci were selected to determine an individual’s hybrid class. Loci considered most informative were shown to have the greatest amount of divergence (*F*_ST_) between the parental populations. This was quantified using Arlequin Version 3.5 [57], where per locus *F*_ST_ values of the founder populations were calculated using default settings as part of the loci under selection method without the hierarchical island model. This dataset was then filtered manually in R, selecting the top 300 loci with an *F*_ST_ value equal to one. NEWHYBRIDS was then used to determine the hybrid class of each individual with a genotype frequency specified as; pure-bred Barrow Island (BWI: 1.0/0.0/0.0/0.0), pure-bred Dryandra (DRY: 0.0/0.0/0.0/1.0), F_1_ hybrid (F_1_: 0.0/0.5/0.5/0.0), F_2_ hybrid (F_2_: 0.25/0.25/0.25/0.25), backcross to pure-bred Barrow Island (F_1_×BWI: 0.5/0.25/0.25/0.0) and backcross to pure-bred Dryandra (F_1_×DRY: 0.0/0.25/0.25/0.5) [69]. Results were replicated five times to ensure consistency at 100,000 MCMC sweeps following a burn-in period of 10,000 with uninformative priors (Jeffreys) given to both allele frequency and admixture distributions. A posterior probability value of 0.8 was used as a threshold to assign individuals to different hybrid classes. Only one individual fell below this threshold and was assumed to belong to an advanced hybrid class after F_2_. Since we would expect more than a single individual to belong to an advanced hybrid class after eight years, analysis was re-run using a dataset with a MAF of 0.2 (4995 SNPs) and 0.4 (1645 SNPs) to exclude rare alleles and increase the likelihood of detecting differences between hybrid classes [70]. Results were consistent with the dataset using a MAF of 0.05 and therefore advanced hybrid classes after F_2_ could not be detected in this study. This is likely a result of the NEWHYBRIDS program unable to detect the differentiation in interclass heterozygosity in these advanced hybrid generations [69,71,72]. The single individual that fell below the 0.8 threshold was excluded from further analyses. 

To determine the impact of ancestry on fitness parameters (apparent survival and per capita recruitment rate) within the Matuwa population we used the R package openCR [73]. OpenCR fits nonspatial models of the Cormack-Jolly Seber (CJS) and Jolly-Seber-Schwarz-Arnason (JSSA) types. The JSSA models each have several parametrizations of recruitment and can be offered in both full and conditional likelihood forms based on the number of detected individuals (see [73] for more information). Apparent survival is defined as the probability that a marked animal at occasion t survives until occasion t + 1 (i.e., between primary trapping sessions). One of the major limitations of survival estimates using capture-mark-recapture data is the inability to distinguish between mortality and emigration. Because individuals in this population are unable to disperse due to the fenced enclosure, survival estimates in this study are able to directly reflect mortality. Per capita recruitment is the probability of new animals at occasion t entering the marked population between occasion t and t + 1 and, therefore, provides an indication of reproductive success as the only potential source of new individuals within the closed translocated population is through reproduction. 

Several reduced models were tested in openCR to determine which model was the best to estimate fitness proxies. The JSSA models with all recruitment parametrizations and the CJS model were tested to determine the best model to estimate apparent survivorship. JSSA models parameterized in terms of per capital recruitment (*f*) were tested to determine the best model to estimate recruitment. All JSSA models were fitted with both full and conditional likelihood forms. Models were compared using corrected AIC values with the best fit model selected based on the lowest AIC value [74]. A CJS model was selected to predict survivorship estimates and a JSSA model fitted with conditional likelihood was selected to calculate recruitment estimates (Table S2). Hybrid class and sex were then added as covariates in these models. Sex was excluded from estimates of recruitment due to very high error margins and the inability of the model to accurately determine recruitment for each sex within each hybrid class. Estimates, standard errors, and 95% confidence intervals were calculated per primary trapping session and were averaged per hybrid class. All founder individuals (BWI *N* = 67, DRY *N* = 87) were used for these analyses. Differences between covariate groups (i.e., hybrid classes and sex) were determined using a Friedman’s ANOVA paired by trapping session.

## 3. Results

### 3.1. Parameter Optimization for SNP Genotyping

The systematic evaluation of STACKS parameters, following Paris et al. [50] and Mastretta-Yanes et al. [51], identified optimal parameters for *B. lesueur*. Paris et al. [50] recommended aiming for coverage thresholds greater than 25×, which improves the robustness of the dataset to variation in sequence quality. The mean coverage per locus was lowest when *m* =3 and increased as *m* increased (Figure S1). The number of assembled loci, polymorphic loci, and SNPs all decreased as *m* increased (Figure S2). The number of polymorphic loci and number of SNPs was greatest at *m* = 3. However, *m* = 3 also had the highest incidence of erroneous loci, allele and SNP calls between replicated samples (Table S3). Therefore, *m* was set to four to decrease the number of errant reads and increase the coverage across the dataset, while also calling a reasonable number of polymorphic loci and SNPs. Changing the *M* parameter had very little impact on the number of assembled loci or on the rate of errant reads (Figure S2 and Table S3). The optimal *M* value was set to three, as this had the highest number of polymorphic loci and SNPs, and the lowest error rates. These parameters resulted in a call rate of 21,267 SNPs with an error rate of 0.0128 in the replicated samples.

### 3.2. Effects of Admixing on Gentic Diversity

A total of 12,347 SNPs (80% complete) was used to assess genetic diversity and investigate the genetic structure of *B. lesueur* at Matuwa. The admixed Matuwa population showed significant differences in all genetic diversity parameters relative to either founder population (Table 2). Pairwise tests between the Barrow Island and each year of the translocated population revealed statistically significant differences (χ^2^ = 9247.7, *p* < 0.001 and χ^2^ = 8436.6, *p* < 0.001 for allelic richness and observed heterozygosity respectively). Similarly, Dryandra and each year of the translocated population also showed significant differences (χ^2^ = 8344.6, *p* < 0.001 and χ^2^ = 7671.1, *p* < 0.001 for allelic richness and observed heterozygosity respectively). The two founder populations were also found to have statistically significant differences in all genetic diversity parameters, with genetic diversity (allelic richness and observed heterozygosity) being greater in the individuals from Barrow Island relative to the Dryandra individuals (Wilcoxon rank sum tests, *p* < 0.001). In the admixed Matuwa population genetic diversity peaked in 2015 and then slightly decreased in later years (Table 2). MLH was used to determine differences in heterozygosity between sexes with males found to have significantly higher MLH in comparison to females in the founder populations (Wilcoxon rank sum tests, *p* < 0.05; Table S4); however, this was not evident in the translocated population (Wilcoxon rank sum tests, *p* > 0.05). 

Multi-locus F*_IS_* values from both founder populations and all years in the translocated Matuwa population were found to be significantly different from zero (Table 2 and Table S5). Average F*_IS_* values from Barrow Island and Dryandra samples were significantly different (Wilcoxon rank sum test, *p* < 0.001) and were both significantly different to the translocated population (χ^2^ = 2975.6, *p* < 0.001 and χ^2^ = 2510.8, *p* < 0.001 respectively). Average F*_IS_* values for the Matuwa samples typically decreased over time.

### 3.3. Population Structure of Bettongia Lesueur at Matuwa

Pairwise *F*_ST_ estimates show significant divergence between the two founder groups (*F*_ST_ = 0.615, *p* < 0.001, Table 3). There was also significant genetic differentiation between the translocated population and both founder populations, with all collection years being significantly different from Dryandra (*p* < 0.001) and all collection years except for 2010 and 2011 significantly different from Barrow Island (*p* < 0.001, Table 3). Pairwise *F*_ST_ estimates show allele frequencies in the translocated population to become more similar to Barrow Island over time and generally diverged over time in comparison to Dryandra. There were no significant temporal changes in allele frequencies within the translocated population except for 2010, which was significantly different to all collection years except for 2011 (*F*_ST_ = 0.058, *p* < 0.05) and 2012 (*F*_ST_ = 0.167, *p* < 0.05). This may be due to the small sample size of 2010 with only four individuals. 

**Figure 2:**
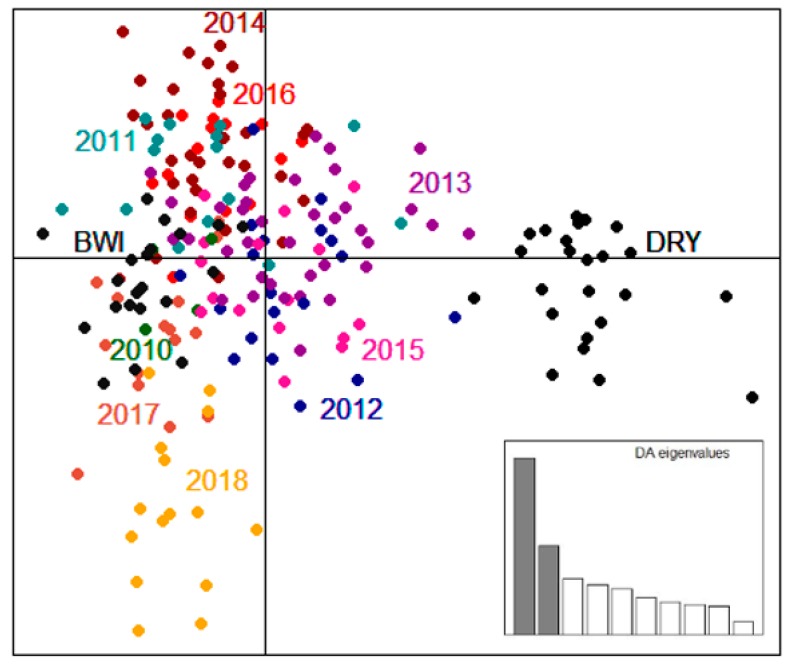
Discriminant Analysis of Principal Components (DAPC) of *Bettongia lesueur* illustrating the relationship between the founding groups, Barrow Island (BWI: dark grey) and Dryandra (DRY: black) and the Matuwa translocated population between 2010 and 2018 based on retaining 90% of cumulative variance. Each dot represents an individual colored by population. The inset shows the histogram of discriminant analysis eigenvalues.

Consistent with the pairwise *F*_ST_ values (Table 3), DAPC results also show that the two founder populations are genetically distinct and that the translocated population appears to be more genetically similar to Barrow Island than Dryandra (Figure 2). Interestingly, samples from 2013 and 2015 in the Matuwa translocated population were among the most genetically diverse (Table 3) and were observed to be the population most intermediate between the two founding groups. 

**Figure 3:**
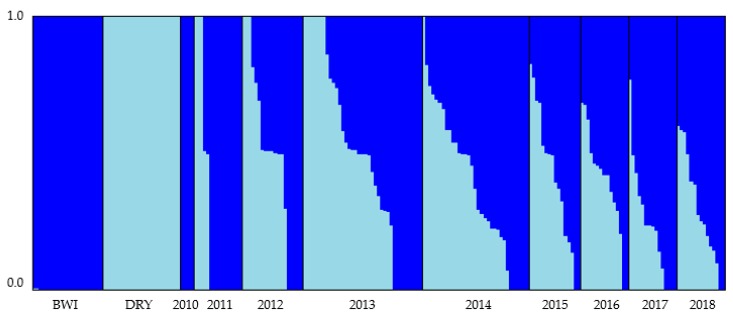
Summary of the clustering results from fastSTRUCTURE for the Matuwa translocated population of *Bettongia lesueur* assuming two admixed populations (*K* = 2). Each individual is represented by a bar showing the individual’s estimated membership to a particular cluster (represented by different colors). Black lines separate samples from each of the founder populations (Barrow Island and Dryandra) and between years.

Both the programs fastSTRUCTURE and gghybrid gave consistent results for the Bayesian clustering analysis (Table S6) with 31% of individuals classed as pure Barrow Island (Q-value ≥ 0.99), 51% classed as hybrids (Q-value ranged between 0.14 and 0.93) and 18% classed as pure Dryandra (Q-value ≤ 0.01). Bayesian clustering analysis on the Matuwa translocated population also revealed the distinct differences between the founder populations and changes in the genetic composition of the translocated population over time (Figure 2). Consistent with the pairwise *F*_ST_ values (Table 3), membership to the cluster representing Barrow Island became more prominent over time (Figure 3). Genetic mixing was evident within the population across all years except for 2010. After 2015, all individuals were either most similar to Barrow Island or intermediate between the two groups, and there were no individuals that were considered most similar to Dryandra. Genetic introgression in the years 2016, 2017, and 2018 appear to have stabilized as the proportion of either cluster remains relatively consistent between these years (Figure 3). 

Consistent with the general trend of Barrow Island becoming more prominent in the Matuwa population over time (Table 3, Figure 2 and Figure 3), allele frequencies fixed for Barrow Island ancestry were found to generally be increasing over time and allele frequencies fixed for Dryandra ancestry generally decreasing over time (Figure S3). 

### 3.4. Impact of Admixing on Fitness

A total of 8182 SNPs (95% complete) were used to determine ancestry of all the progeny born at Matuwa that had a posterior probability above 0.8 in NEWHYBRIDS (*N* = 169); 27% were classed as pure-bred Barrow Island, 23% were backcrossed to Barrow Island, 17% were classed as F_1_ hybrids, 14% were classed as F_2_ hybrids, 11% were classed as backcrossed to Dryandra and only 8% were designated as pure-bred Dryandra individuals. 

**Figure 4:**
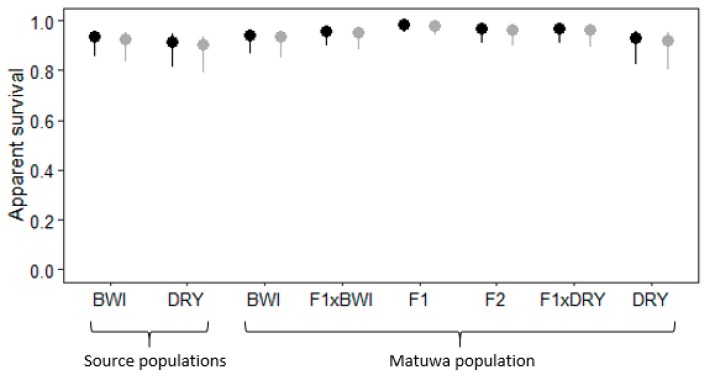
Average apparent survival with 95% confidence intervals for female (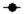) and male (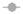) *Bettongia lesueur* individuals from founder populations; Barrow Island and Dryandra and each hybrid class of the translocated population; pure-bred Barrow Island (BWI), pure-bred Dryandra (DRY), backcross to Barrow Island (F_1_×BWI), F_1_ hybrid (F_1_), F_2_ hybrid (F_2_), backcross to Dryandra (F_1_×DRY) and pure-bred Dryandra (DRY).

Apparent survival for both founder groups and the Matuwa population were all relatively high (Figure 4). There were no statistically significant differences in survivorship between sexes within each group (χ^2^ = 390, df = 15, *p* > 0.05) and as expected, apparent survival was highest in the F_1_ hybrids. Both female and male F_1_ hybrids had statistically higher survival than all groups (*p* < 0.01) except F_2_ hybrids and individuals back-crossed to Dryandra (F_1_×DRY) (Tables S7 and S8). F_2_ hybrids also had significantly higher apparent survival than all groups (*p* < 0.01) except for F_1_ hybrids and individuals backcrossed to either Barrow Island (F_1_×BWI) or Dryandra (F_1_×DRY). There was no statistically significant difference in survival between founder populations, however, male Barrow Island founders had significantly lower survival than male individuals backcrossed to Barrow Island (F_1_×BWI) (*p* < 0.05) and female Dryandra founders had significantly lower survival than pure-bred Barrow Island females from the Matuwa population (*p* < 0.01; Table S8). Furthermore, pure-bred individuals (BWI and DRY) tended to have significantly lower apparent survival rates than admixed individuals in the Matuwa population; pure-bred Barrow Island and pure-bred Dryandra individuals had significantly lower survival rates than F_1_ hybrids, F_2_ hybrids and individuals backcrossed to Dryandra (F_1_×DRY) (*p* < 0.05), and pure-bred Dryandra individuals also had significantly lower survival rates than individuals that were backcrossed to Barrow Island (F_1_×BWI) (*p* < 0.01; Table S8). 

**Figure 5:**
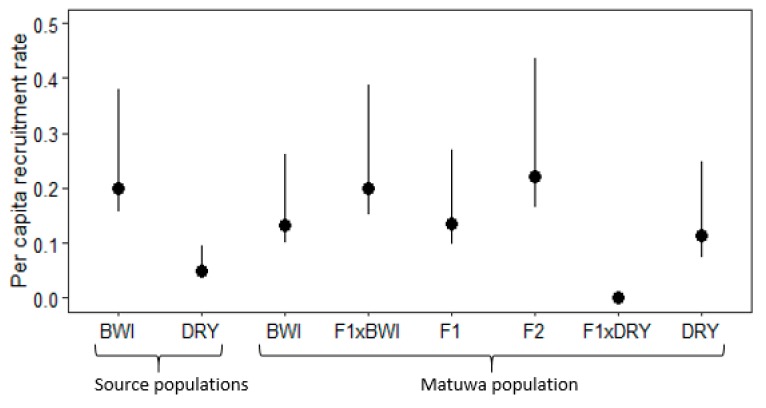
Average per capita recruitment rate with 95% confidence intervals for *Bettongia lesueur* individuals from founder populations; Barrow Island and Dryandra and each hybrid class of the translocated population; pure-bred Barrow Island (BWI), pure-bred Dryandra (DRY), backcross to Barrow Island (F_1_×BWI), F_1_ hybrid (F_1_), F_2_ hybrid (F_2_), backcross to Dryandra (F_1_×DRY) and pure-bred Dryandra (DRY).

Recruitment between founder populations was found to be significantly different with Barrow Island founders having a higher recruitment (χ^2^ = 182, df = 7, *p* < 0.01; Figure 5, Tables S7 and S9). Interestingly, F_2_ hybrids were found to have the highest recruitment rate and were significantly higher to all groups (*p* < 0.01) apart from Barrow Island founders and individuals backcrossed to Barrow Island ancestry (F_1_×BWI). Although F_1_ hybrids did not have as high recruitment as F_2_ hybrids, recruitment was still significantly higher than individuals backcrossed to Dryandra (F_1_×DRY) (*p* < 0.01) and DRY founders (*p* < 0.01; Table S9). Individuals with Dryandra ancestry generally had the lowest recruitment rate, with Dryandra founders having significantly lower recruitment than all groups (*p* < 0.01) except pure-bred Barrow Island (BWI) and Dryandra (DRY) individuals as well as individuals backcrossed to DRY (F_1_×DRY). Individuals backcrossed to Dryandra (F_1_×DRY) were found to have no recruitment within the population.

## 4. Discussion

The translocated population of *B. lesueur* at Matuwa offered a rare opportunity to investigate the genetic and demographic consequences of admixing two genetically distinct subspecies across several generations of outcrossing. Using high-resolution genomic markers through ddRAD sequencing, this study showed that the two founder populations are genetically distinct and readily interbreed resulting in an increase in genetic diversity with no negative effects on survivorship or reproductive capacity. 

### 4.1. Influence of Admixing on Genetic Diversity

The immigration of new individuals into small inbred populations is predicted to improve genetic diversity and decrease the proportion of individuals that are homozygous for alleles identical by descent [24,26]. Similarly, mixing multiple sources in a translocation, even if from inbred populations, is expected to have the same effect [19]. This was evident in the admixed Matuwa population of *B. lesueur* as genetic diversity (allelic richness, heterozygosity, and multi-locus heterozygosity) was observed to be higher relative to both founder populations, potentially resulting in improved adaptive potential for this population to respond to environmental changes. This increase in genetic diversity is a direct result of mixing the two genetically distinct subspecies, which have been isolated for a relatively long period of time thereby different sets of alleles having occurred as a result of genetic drift [75,76]. Furthermore, this introgressed genetic diversity between subspecies has been maintained over a period of eight years consistent with previous studies quantifying the long-term positive benefits of admixing source populations [9,77,78], as was demonstrated in the admixed Arid Recovery population of this species at Roxby Downs [78]. This population was established from two subpopulations consisting of the Shark Bay subspecies (from Bernier and Dorre Island) and found increased genetic diversity relative to the founder individuals that was maintained over 18 years [78]. The significant F*_IS_* values of all years in the translocated population may be reflective of mating between individuals with difference ancestries to be non-random [45]. However, this appeared to have little impact on the overall improved genetic diversity of this admixed population. Although our study is limited by the small sample size of some collection years, the success of mixing of source populations in this species provides further support for use of admixture as a useful tool in the conservation of other threatened species [7,9,21,24,77,78].

Population structure analyses indicated genetic differentiation between the Matuwa translocated population and their founding groups. The translocated population was initially significantly divergent from both founder populations and this pattern changed over time as the translocated population became more genetically similar to the Barrow Island population. This could be due to selection favoring Barrow Island alleles over Dryandra alleles (i.e., from climate and environmental conditions), from demographic effects, such as social structuring within the population [59,79], the influence of density-dependent and density-independent mechanisms on life history traits (i.e., body size and reproductive rate [80,81], or endogenous genetic barriers as a result of intrinsic incompatibilities between the two subspecies [82,83]. Genetic drift is also a possible explanation for the differentiation observed between founder populations as random changes in allele frequencies in the isolated source populations may result in genetic differentiation over generations [36]. However, this is a less likely explanation for the Matuwa population due to the relatively large population size of over 900 individuals [47] and the relatively short period of time since the translocated population has been established. More research is required to fully understand how drift and selection can be distinguished and their relative roles in subpopulation structure, particularly in the context of translocations when animals are moved between different climatic zones and environmental conditions [78]. 

Within the Matuwa population, ancestry proportions were variable across individuals and through time. The four individuals captured in 2010 were found to be of Barrow Island ancestry and were trapped at approximately similar times to when the Dryandra founders were released. Admixed individuals were present in 2011, the first year after all founders were initially released, and by 2015 almost all individuals were hybrids. In later years, there was a clear bias towards the Barrow Island ancestry despite only 67 founding individuals being released in comparison to the 87 Dryandra individuals. This pattern of a higher proportion of ancestry from one founder in a mixed population was also observed at Roxby Downs [78]. It was thought that this bias at Roxby Downs could be due to one founder being released before the other allowing the first group to acclimatize to the new habitat, establish social groups and secure preference in any pre-existing burrows thereby having an advantage over the second group. This is also a possibility at Matuwa as founder individuals were released incrementally with Barrow Island individuals released in February and the majority of Dryandra individuals released in August and October. Furthermore, Thavornkanlapachai [45] showed the Matuwa population to be undergoing asymmetrical introgression due to crosses between smaller-sized Barrow Island males and larger-sized Dryandra females being more common than expected. Therefore, Barrow Island males are thought to be able to reproduce with all females whereas Dryandra males can only contribute offspring from Dryandra females. This provides an explanation for the bias towards the Barrow Island individuals and this trend may be further continuing. 

### 4.2. Impact of Ancestry and Genetic Mixing on Fitness Traits 

Genetic admixing can increase the fitness of individuals through heterosis [20,26]. This was observed in the improved survivorship of both male and female F_1_ and F_2_ hybrids. The improved survival of F_1_ and F_2_ hybrids likely reflects heterozygosity masking the expression of deleterious recessive alleles (dominance effects), heterozygote advantage (overdominance) or possibly positive epistasis, whereby recombination of new genotypes provides a favorable interaction enabling higher survival rates and subsequent fitness [20,84]. Further, F_2_ hybrids had significantly improved recruitment relative to all other hybrid classes, except for Barrow Island founders and individuals backcrossed to Barrow Island ancestry (F_1_×BWI), and although this was not observed in the F_1_ generation, this effect may also be explained by positive epistasis or the action of complementary genes where the combination of alleles with additive effects from each of the parental populations results in a complementary interaction [85].

While individuals with Barrow Island ancestry were found to have higher survival and recruitment in comparison to individuals with Dryandra ancestry, hybrid generation classes were not found to be statistically different (except recruitment rate of Dryandra founders to all other groups). Individuals backcrossed to Dryandra ancestry were found to have no recruitment within the population. Nearly all (95%) of individuals from this hybrid class were trapped prior to 2017, and since sexual maturity is expected to be reached within a year, we would expect to detect some recruitment. The observed recruitment bias may reflect intrinsic incompatibilities between source populations becoming apparent only when hybrid individuals have a high proportion of Dryandra ancestry. However, given the low recruitment of individuals with a pure Dryandra ancestry, and the consistently high recruitment of other hybrid classes, it is more likely that this reflects an overall lower recruitment rate of the Dryandra population lineage. Although morphology was not investigated in this study, Thavornkanlapachai [45] demonstrated an underlying genetic basis to the differences in body size of the two founding groups and consequently fitness differences between small and large morphologies may also lead to genetic changes within the Matuwa population if a particular morphological trait was selectively favored. Further investigating the genetic basis of morphological differences and the possible loci under selection would provide insight into possible advantages in differing morphology and the degree to which morphological traits might influence other fitness parameters such as survival and recruitment. Further research is also required to understand the impact of mating strategies, sex ratio and sex-specific reproductive skews on both the maintenance of genetic diversity as well as fitness traits [78]. This is vital to understanding the basic ecology of natural populations and is also critical to improving the success of reintroductions and translocations. Little is known about the mating strategies and behavioral traits of *B. lesueur* and how this may differ between subspecies and in novel environments. Therefore, it is difficult to predict the behavioral consequences when admixing populations and how this may lead to assortative mating within the translocated population and the subsequent impact on genetic diversity and fitness. 

### 4.3. Implications for Conservation and Management 

These results emphasize the positive impact admixing multiple sources can have on genetic diversity with no apparent fitness consequences or outbreeding depression indicated for *B. lesueur* to date. Previous studies have demonstrated that admixture of diverged populations substantially increases genetic diversity in reintroduced populations, even when divergence between populations was low [3,9,77,78,86]. Here, we demonstrate that highly diverged populations can have the same benefits. Admixture as a conservation tool for translocations is infrequently utilized due to concerns about outbreeding depression and there are multiple guidelines to predict the relative risks of outbreeding depression from genetic admixing [27,28]. However, as few as 1–10 migrants per generation may be all that is necessary to prevent the loss of genetic diversity and adaptive potential [87], and therefore the possibility of outcrossing as an effective management option is highly beneficial in conserving endangered species when used appropriately. This is particularly advantageous for threatened species where individuals are of low abundance, and therefore provides an alternative by establishing and maintaining populations with relatively few individuals while still maintaining genetic diversity.

While no negative consequences were found in regards to survivorship, there is concern in regard to the lack of recruitment of individuals backcrossed to Dryandra ancestry and how this may be contributing to the suspected continued asymmetrical introgression. This generation class and Dryandra founders were the only group to have lower recruitment and therefore lower fitness, and the genetic mechanisms behind this trend remain largely unknown. Regardless of whether admixture results in improved fitness, the mixing of multiple source populations will decrease the risks of inbreeding depression. This study further highlights the importance of genetic monitoring over multiple generations (F_2_ and beyond) to adequately evaluate the outcomes of admixing and the long-term viability of translocated populations.

## Figures and Tables

**Table 1:** Translocation history and the release of the founding individuals for *Bettongia lesueur* at Matuwa. Subspecies include Shark Bay subspecies (*Bettongia lesueur lesueur*) and Barrow Island subspecies (*Bettongia lesueur* unnamed subspecies). Animal origin includes individuals from Dryandra (DRY) and Barrow Island (BWI).

Subspecies	Animal Origin	Number of individuals Released	Month of Release
*Bettongia lesueur lesueur*	DRY	20	January
*Bettongia lesueur* unnamed subspecies	BWI	67	February
*Bettongia lesueur lesueur*	DRY	80	August
*Bettongia lesueur lesueur*	DRY	9	October

**Table 2:** Sample sizes, estimates of genetic variation, and inbreeding in the founder and translocated population of *Bettongia lesueur*.

Population	N	A*_R_*	H*_e_*	H*_o_*	F*_IS_*
**Founder populations**				
Barrow Island	22	1.549 (0.004)	0.209 (0.002)	0.196 (0.002)	0.050 *
Dryandra	24	1.452 (0.004)	0.179 (0.002)	0.177 (0.002)	0.012 *
**Translocated population**				
2011	15	1.916 (0.002)	0.334 (0.001)	0.238 (0.001)	0.250 *
2012	19	1.929 (0.001)	0.356 (0.001)	0.326 (0.001)	0.072 *
2013	37	1.930 (0.001)	0.354 (0.001)	0.303 (0.001)	0.121 *
2014	33	1.924 (0.001)	0.346 (0.001)	0.327 (0.001)	0.049 *
2015	16	1.930 (0.001)	0.351 (0.001)	0.343 (0.002)	0.019 *
2016	15	1.927 (0.002)	0.350 (0.001)	0.335 (0.002)	0.037 *
2017	15	1.890 (0.002)	0.316 (0.001)	0.302 (0.002)	0.040 *
2018	15	1.911 (0.002)	0.332 (0.001)	0.315 (0.002)	0.042 *

N is the number of individuals sampled, A*_R_* is allelic richness, H*_e_* is the expected heterozygosity under HWE, H*_o_* is the observed heterozygosity and F*_IS_* is the inbreeding coefficient. Standard errors are in parentheses. F*_IS_* estimates significantly greater than zero after correction for multiple comparisons are denoted with an asterisk (*).

**Table 3:** Pairwise *F*_ST_ estimates between founder populations of *Bettongia lesueur*, Barrow Island (BWI) and Dryandra (DRY), and samples taken from the Matuwa translocated site between 2010 and 2018 for SNP data.

	BWI	DRY	2010	2011	2012	2013	2014	2015	2016	2017	2018
BWI	-										
DRY	0.615 *	-									
2010	0.018	0.632 *	-								
2011	0.061	0.410 *	0.058	-							
2012	0.191 *	0.246 *	0.167	0.025	-						
2013	0.179 *	0.229 *	0.164 *	0.028	−0.007	-					
2014	0.126 *	0.302 *	0.112 *	0.001	0.002	0.004	-				
2015	0.156 *	0.311 *	0.137 *	0.005	−0.005	−0.004	−0.007	-			
2016	0.143 *	0.322 *	0.120 *	0.0005	−0.004	0.003	−0.004	−0.009	-		
2017	0.055 *	0.441 *	0.046 *	−0.010	0.045	0.046	0.016	0.020	0.017	-	
2018	0.089 *	0.395 *	0.080 *	−0.010	0.021	0.025	0.005	0.005	0.004	0.001	-

*F*_ST_ estimates significantly greater than zero (*p* < 0.05) after correction for multiple comparisons are denoted with an asterisk (*).

## Supplementary Materials

The following are available online at https://www.mdpi.com/2073-4425/10/11/851/s1. Figure S1. Coverage for all individuals of *Bettongia lesueur* sampled (including founder individuals) represented as a box plot, where mean coverage (white) is the average coverage for primary reads and mean merged coverage (grey) is the coverage after merging alleles. Average is indicated by a red diamond. Middle horizontal line reflects the median, the boxes are bound by the 25th and 75th quartiles and the vertical lines show the minimum and maximum range of values, Figure S2. Plots of iterating values for the minimum number of identical raw reads required to make a stack (*m*) and the minimum number of mismatches allowed between loci when processing a single individual (*M*), evaluating the effects of varying STACKS parameters on: the number of assembled loci; the number of polymorphic loci; the number of SNPs. Middle horizontal line reflects the median, the boxes are bound by the 25th and 75th quartiles and the vertical lines show the minimum and maximum range of values. Boxplots also depict the average values (red dot) for the total 228 individuals (including replicates) sampled from Matuwa, Figure S3. Average allele frequency ± standard error in the admixed *Bettongia lesueur* population at Matuwa over time for 954 loci with fixed alleles for either Barrow Island founders (BWI) or Dryandra (DRY) founders, Table S1. Number of *Bettongia lesueur* samples from Matuwa selected for genetic analysis. All individuals were born at Matuwa. ‘Caught early’ individuals were first trapped between 2010 and 2013 and ‘caught later’ individuals were first trapped between 2014 and 2018. Long lived individuals had four or more years of capture histories and short lived individuals had less than four years of capture histories, Table S2. AIC values for reduced Cormack-Jolly-Seber (CJS) and Jolly-Seber-Schwarz-Arnason (JSSA) models tested using the R package openCR for apparent survival and per capita recruitment rate estimates of *Bettongia lesueur* at Matuwa, Table S3. Average error rates of replicated samples for iterating STACKS parameters of *m* (minimum number of raw reads required to form a stack) and *M* (distance allowed between two stacks). Standard errors are in parentheses, Table S4. Multi-locus heterozygosity (MLH) for females and males in either founder population, Barrow Island (BWI) and Dryandra (DRY), and the translocated Matuwa population. Standard errors are stated in parentheses. Table S5. Upper and lower 95% confidence intervals calculated by bootstrapping for FIS values for Barrow Island (BWI), Dryandra (DRY) and the Matuwa translocated population between 2011 and 2018. Table S6. Estimated membership to a particular cluster assuming two admixed populations for each individual from the founder populations (BWI and DRY) and the translocated population from 2010 to 2018 estimated using fastSTRUCTURE and gghybrid. Hybrid class determined using NEWHYBRIDS is also shown for the translocated population: pure-bred Barrow Island (BWI), pure-bred Dryandra (DRY), backcross to Barrow Island (F_1_×BWI), F_1_ hybrid (F_1_), F_2_ hybrid (F_2_), backcross to Dryandra (F_1_×DRY) and pure-bred Dryandra (DRY). Table S7. Apparent survival and recruitment rate estimates ± standard error for founding individuals of *Bettongia lesueur* from each founder population and each hybrid class of the translocated population: pure-bred Barrow Island (BWI), pure-bred Dryandra (DRY), backcross to Barrow Island (F_1_×BWI), F_1_ hybrid (F_1_), F_2_ hybrid (F_2_), backcross to Dryandra (F_1_×DRY) and pure-bred Dryandra (DRY). 95% confidence intervals are in parentheses. Table S8. Friedman’s ANOVA p-values for male and female apparent survival estimates of *Bettongia lesueur* from founder populations; Barrow Island and Dryandra and each hybrid class of the translocated population; pure-bred Barrow Island (BWI), pure-bred Dryandra (DRY), backcross to Barrow Island (F1×BWI), F1 hybrid (F1), F2 hybrid (F2), backcross to Dryandra (F1×DRY) and pure-bred Dryandra (DRY). Significant *p*-values (*p* < 0.05) are denoted with an asterisk. Table S9. Friedman’s ANOVA *p*-values for estimates of per capita recruitment rate of *Bettongia lesueur* from founder populations; Barrow Island and Dryandra and each hybrid class of the translocated population; pure-bred Barrow Island (BWI), pure-bred Dryandra (DRY), backcross to Barrow Island (F_1_×BWI), F_1_ hybrid (F_1_), F_2_ hybrid (F_2_), backcross to Dryandra (F_1_×DRY) and pure-bred Dryandra (DRY). Significant *p*-values (*p* < 0.05) are denoted with an asterisk.

SNP dataset vcf file from STACKS pipeline and the capture-mark-recapture history of *Bettongia lesueur* at Matuwa for openCR analysis can be found at http://dx.doi.org/10.17632/vg983472v3.1.
