# Supplementary material for: Population Genomics of Bettongia lesueur: Admixing Increases Genetic Diversity with no Evidence of Outbreeding Depression
Source: Genes (Basel). 2019 Oct 28;10(11):851. doi: 10.3390/genes10110851 (PMC6896034; doi:10.3390/genes10110851)
Supplement: Supplementary file 1 [file genes-10-00851-s001.zip › Figure S1. Coverage for all individuals of Bettongia lesueur sampled.pdf]

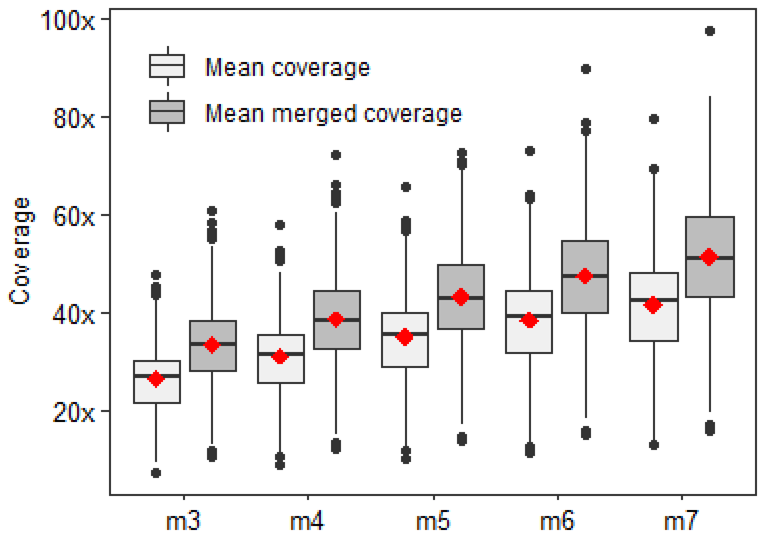

Figure S1. Coverage for all individuals of *Bettongia lesueur* sampled (including founder individuals) represented as a box plot, where mean coverage (white) is the average coverage for primary reads and mean merged coverage (grey) is the coverage after merging alleles. Average is indicated by a red diamond. Middle horizontal line reflects the median, the boxes are bound by the 25th and 75th quartiles and the vertical lines show the minimum and maximum range of values.
