# Supplementary material for: Population Genomics of Bettongia lesueur: Admixing Increases Genetic Diversity with no Evidence of Outbreeding Depression
Source: Genes (Basel). 2019 Oct 28;10(11):851. doi: 10.3390/genes10110851 (PMC6896034; doi:10.3390/genes10110851)
Supplement: Supplementary file 1 [file genes-10-00851-s001.zip › Figure S2. Plots of iterating values for m and M in STACKS pipeline.pdf]

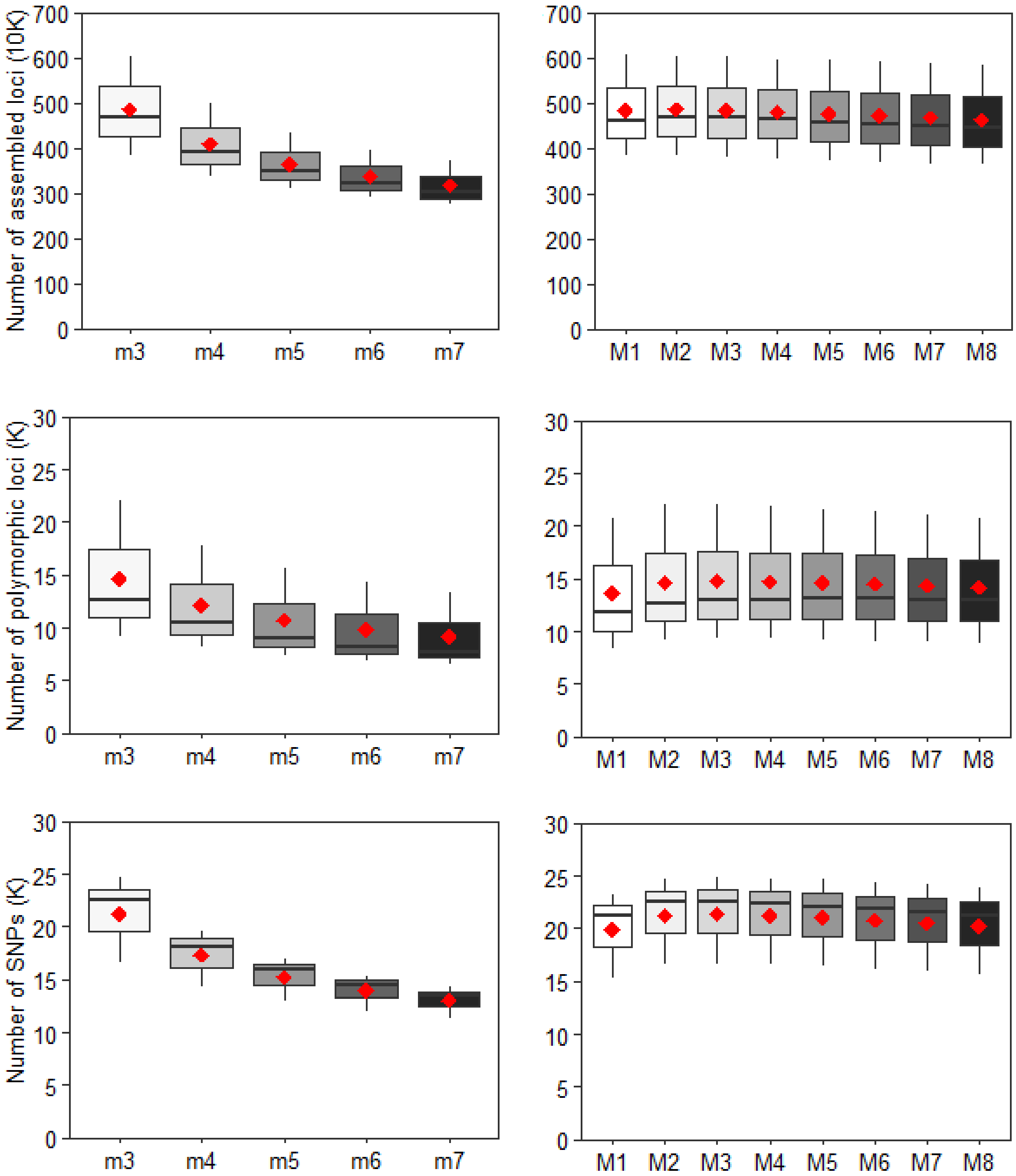

Figure S2. Plots of iterating values for the minimum number of identical raw reads required to make a stack (m) and the minimum number of mismatches allowed between loci when processing a single individual (M), evaluating the effects of varying STACKS parameters on: the number of assembled loci; the number of polymorphic loci; the number of SNPs. Middle horizontal line reflects the median, the boxes are bound by the 25th and 75th quartiles and the vertical lines show the minimum and maximum range of values. Boxplots also depict the average values (red dot) for the total 228 individuals (including replicates) sampled from Matuwa.
