# Supplementary material for: Population Genomics of Bettongia lesueur: Admixing Increases Genetic Diversity with no Evidence of Outbreeding Depression
Source: Genes (Basel). 2019 Oct 28;10(11):851. doi: 10.3390/genes10110851 (PMC6896034; doi:10.3390/genes10110851)
Supplement: Supplementary file 1 [file genes-10-00851-s001.zip › Figure S3. Average allele frequency.pdf]

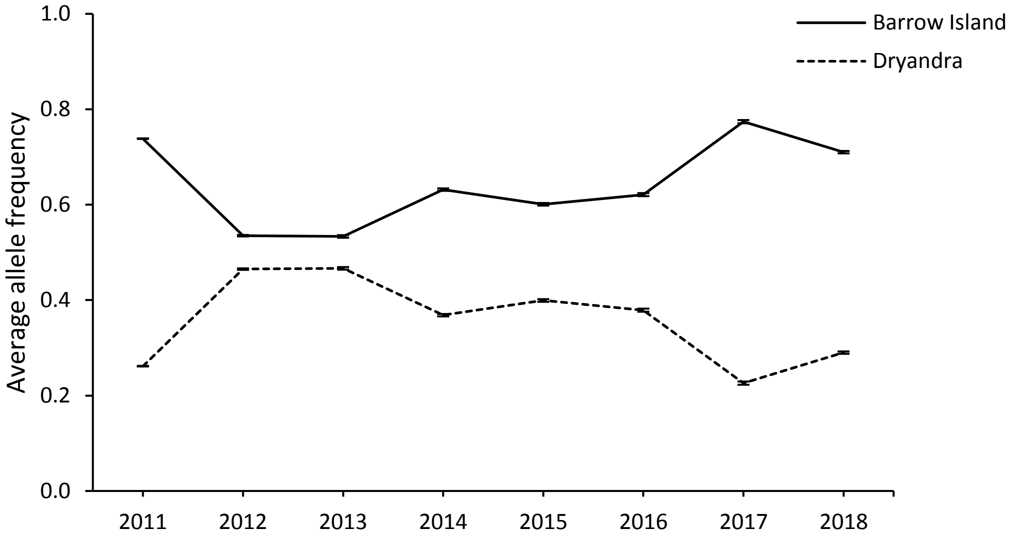

Figure S3. Average allele frequency ± standard error in the admixed B*ettongia lesueur* population at Matuwa over time for 954 loci with fixed alleles for either Barrow Island founders (BWI) or Dryandra (DRY) founders.
