# Supplementary material for: Population Genomics of Bettongia lesueur: Admixing Increases Genetic Diversity with no Evidence of Outbreeding Depression
Source: Genes (Basel). 2019 Oct 28;10(11):851. doi: 10.3390/genes10110851 (PMC6896034; doi:10.3390/genes10110851)
Supplement: Supplementary file 1 [file genes-10-00851-s001.zip › Table S2. AIC values for reduced models tested in openCR.pdf]

Table S2. AIC values for reduced Cormack-Jolly-Seber (CJS) and Jolly-Seber-Schwarz-Arnason (JSSA) models tested using the R package openCR for apparent survival and per capita recruitment rate estimates of *Bettongia lesueur* at Matuwa.

Model type	Likelihood	Model parametrizations	logLik	AIC	AICc	dAIC
CJS		p~1 phi~1	-9074.23	18152.46	18152.48	0
JSSA	Conditional likelihood	p~1 phi~1 b~1	-15053.3	30112.58	30112.62	11960.12
JSSA	Conditional likelihood	p~1 phi~1 f~1	-14939.6	29885.22	29885.26	11732.76
JSSA	Conditional likelihood	p~1 phi~1 gamma~1	-14939.6	29885.22	29885.26	11732.76
JSSA	Conditional likelihood	p~1 phi~1 lambda~1	-14939.6	29885.22	29885.26	11732.76
JSSA	Full likelihood	p~1 phi~1 b~1 superN~1	-15057.6	30123.28	30123.34	11970.81
JSSA	Full likelihood	p~1 phi~1 f~1 superN~1	-14944	29895.91	29895.97	11743.45
JSSA	Full likelihood	p~1 phi~1 gamma~1 superN~1	-14944	29895.91	29895.97	11743.45
JSSA	Full likelihood	p~1 phi~1 lambda~1 superN~1	-14944	29895.92	29895.98	11743.45
JSSA	Full likelihood	p~1 phi~1 BN~1	-15083.6	30173.16	30173.2	12020.7
JSSA	Full likelihood	p~1 phi~1 N~1	-14993	29991.92	29991.96	11839.46
