# Supplementary material for: Population Genomics of Bettongia lesueur: Admixing Increases Genetic Diversity with no Evidence of Outbreeding Depression
Source: Genes (Basel). 2019 Oct 28;10(11):851. doi: 10.3390/genes10110851 (PMC6896034; doi:10.3390/genes10110851)
Supplement: Supplementary file 1 [file genes-10-00851-s001.zip › Table S3. Average error rates of replicated samples for iterating STACKS parameters.pdf]

Table S3. Average error rates of replicated samples for iterating STACKS parameters of m (minimum number of raw reads required to form a stack) and M (distance allowed between two stacks). Standard errors are in parentheses.

Parameter	Locus error	Allele error	SNP error
**Minimum number of raw reads required to form a stack (m)**			
m3	0.052 (0.040)	0.019 (0.011)	0.019 (0.003)
m4	0.042 (0.035)	0.013 (0.009)	0.013 (0.003)
m5	0.036 (0.031)	0.009(0.008)	0.009 (0.002)
m6	0.034 (0.030)	0.008 (0.007)	0.008 (0.002)
m7	0.033 (0.030)	0.007 (0.007)	0.007 (0.002)
**Distance allowed between two stacks (M)**			
M1	0.053 (0.039)	0.021 (0.011)	0.021 (0.003)
M2	0.052 (0.040)	0.019 (0.010)	0.019 (0.003)
M3	0.052 (0.040)	0.018 (0.010)	0.018 (0.003)
M4	0.052 (0.040)	0.018 (0.010)	0.018 (0.003)
M5	0.053 (0.040)	0.018 (0.010)	0.018 (0.003)
M6	0.053 (0.040)	0.019 (0.010)	0.019 (0.003)
M7	0.053 (0.040)	0.019 (0.011)	0.019 (0.003)
M8	0.053 (0.040)	0.019 (0.011)	0.019 (0.003)
