# Supplementary material for: Population Genomics of Bettongia lesueur: Admixing Increases Genetic Diversity with no Evidence of Outbreeding Depression
Source: Genes (Basel). 2019 Oct 28;10(11):851. doi: 10.3390/genes10110851 (PMC6896034; doi:10.3390/genes10110851)
Supplement: Supplementary file 1 [file genes-10-00851-s001.zip › Table S4. Multi-locus heterozygosity.pdf]

Table S4. Multi-locus heterozygosity (MLH) of *Bettongia lesueur* for females and males in either founder population, Barrow Island (BWI) and Dryandra (DRY), and the translocated Matuwa population. Standard errors are stated in parentheses.

	**Female**	**Male**
**BWI**	0.192 (0.004)	0.202 (0.008)
**DRY**	0.175 (0.004)	0.181 (0.004)
**Matuwa**	0.295 (0.010)	0.326 (0.011)
