# Supplementary material for: Population Genomics of Bettongia lesueur: Admixing Increases Genetic Diversity with no Evidence of Outbreeding Depression
Source: Genes (Basel). 2019 Oct 28;10(11):851. doi: 10.3390/genes10110851 (PMC6896034; doi:10.3390/genes10110851)
Supplement: Supplementary file 1 [file genes-10-00851-s001.zip › Table S8. Friedman's ANOVA p-values for apparent survival.pdf]

Figure S8. Friedman's ANOVA p-values for male and female apparent survival estimates of *Bettongia lesueur* from founder populations; Barrow Island and Dryandra and each hybrid class of the translocated population; pure-bred Barrow Island (BWI), pure-bred Dryandra (DRY), backcross to Barrow Island (F1×BWI), F1 hybrid (F1), F2 hybrid (F2), backcross to Dryandra (F1×DRY) and pure-bred Dryandra (DRY). Significant *p*-values (p<0.05) are denoted with an asterisk.

		Founder populations				*Matuwa population*											
		**BWI**		**DRY**		**BWI**		**F_1 ×BWI**		**F_1**		**F_2**		**F_1×DRY**		**DRY**	
		**F**	**M**	**F**	**M**	**F**	**M**	**F**	**M**	**F**	**M**	**F**	**M**	**F**	**M**	**F**	**M**
Founder population																	
**BWI**	**F**	-															
	**M**	0.697	-														
**DRY**	**F**	0.070	0.999	-													
	**M**	0.006*	0.906	1.000	-												
Matuwa population																	
**BWI**	**F**	1.000	0.168	0.004*	1.600E-04*	-											
	**M**	1.000	0.977	0.358	0.059	0.987	-										
**F_1 ×BWI**	**F**	0.591	0.001*	1.800E-06*	2.200E-08*	0.977	0.168	-									
	**M**	0.963	0.012*	8.800E-05*	1.800E-06*	1.000	0.634	1.000	-								
**F_1**	**F**	8.300E-10*	2.100E-13*	1.500E-13*	1.600E-13*	1.600E-07*	6.800E-12*	0.001*	2.000E-05*	-							
	**M**	9.500E-08*	1.800E-13*	1.400E-13*	1.500E-13*	1.100E-05*	1.200E-09*	0.014*	0.001*	1.000	-						
**F_2**	**F**	3.100E-04*	6.800E-10*	3.600E-13*	1.300E-13*	0.010*	1.100E-05*	0.591	0.145	0.717	0.987	-					
	**M**	0.091	6.600E-06*	7.100E-09*	4.700E-11*	0.525	9.890E-03*	1.000	0.969	0.025	0.207	0.987	-				
**F_1 ×DRY**	**F**	4.800E-06*	1.900E-12*	1.200E-13*	1.300E-13*	3.100E-04*	9.500E-08*	0.115	0.011*	0.993	1.000	1.000	0.655	-			
	**M**	0.006*	5.500E-08*	2.500E-11*	3.000E-13*	0.091	3.100E-04*	0.951	0.547	0.252	0.756	1.000	1.000	0.987	-		
**DRY**	**F**	0.999	1.000	0.717	0.221	0.856	1.000	0.041*	0.285	3.600E-13*	3.800E-11*	6.600E-07*	0.001*	4.000E-09*	2.700E-05 *	-	
	**M**	0.398	1.000	1.000	0.989	0.054	0.856	8.800E-05 *	0.002 *	1.400E-13 *	1.300E-13 *	3.800E-11 *	6.600E-07 *	2.700E-13 *	4.000E-09 *	0.987	-
