# Supplementary material for: Population Genomics of Bettongia lesueur: Admixing Increases Genetic Diversity with no Evidence of Outbreeding Depression
Source: Genes (Basel). 2019 Oct 28;10(11):851. doi: 10.3390/genes10110851 (PMC6896034; doi:10.3390/genes10110851)
Supplement: Supplementary file 1 [file genes-10-00851-s001.zip › Table S9. Friedman's ANOVA p-values for per capita recruitment rate.pdf]

Figure S9. Friedman's ANOVA *p*-values for estimates of per capita recruitment rate of *Bettongia lesueur* from founder populations; Barrow Island and Dryandra and each hybrid class of the translocated population; pure-bred Barrow Island (BWI), pure-bred Dryandra (DRY), backcross to Barrow Island (F_1×BWI), F_1 hybrid (F_1), F_2 hybrid (F_2), backcross to Dryandra (F_1×DRY) and pure-bred Dryandra (DRY). Significant *p*-values (p<0.05) are denoted with an asterisk.

	Founder populations		***Matuwa population***					
	**BWI**	**DRY**	**BWI**	**F_1 ×BWI**	**F_1**	**F_2**	**F_1×DRY**	**DRY**
Founder populations								
**BWI**	-							
**DRY**	2.30E-10*	-						
Matuwa population								
**BWI**	0.0005*	0.1725	-					
**F_1 ×BWI**	0.8863	1.10E-06*	0.0641	-				
**F_1**	0.1158	0.001*	0.7768	0.8627	-			
**F_2**	0.709	7.60E-14*	7.70E-08*	0.0547	0.0003*	-		
**F_1×DRY**	8.30E-14*	0.7768	0.001*	4.80E-11*	4.10E-07*	9.10E-14*	-	
**DRY**	1.40E-06*	0.8627	0.9413	0.001*	0.1158	3.20E-11*	0.0641	-
