# Supplementary material for: Population Genomics of Bettongia lesueur: Admixing Increases Genetic Diversity with no Evidence of Outbreeding Depression
Source: Genes (Basel). 2019 Oct 28;10(11):851. doi: 10.3390/genes10110851 (PMC6896034; doi:10.3390/genes10110851)
Supplement: Supplementary file 1 [file genes-10-00851-s001.zip › Table S1. Number of Bettongia lesueur samples from Matuwa selected for genetic analysis.pdf]

Table S1. Number of *Bettongia lesueur* samples from Matuwa selected for genetic analysis. All individuals were born at Matuwa. Caught early individuals were first trapped between 2010 and 2013 and caught late individuals were first trapped between 2014 and 2018. Long lived individuals had four or more years of capture histories and short lived individuals had less than four years of capture histories.

	Year	Long lived	Short lived	Total
**Caught Early**	2010	3	2	
	2011	8	8	
	2012	11	9	
	2013	24	14	
**Total**		**46**	**33**	**79**
**Caught Late**	2014	23	11	
	2015	11	5	
	2016*	0	15	
	2017*	0	15	
	2018*	0	15	
**Total**		**34**	**61**	**95**

* Years 2016, 2017 and 2018 had no "long lived" individuals due to lack of more than four years of trapping data available and as such were randomly selected (indicated as "short livied" in Table).
