# Supplementary material for: Population Genomics of Bettongia lesueur: Admixing Increases Genetic Diversity with no Evidence of Outbreeding Depression
Source: Genes (Basel). 2019 Oct 28;10(11):851. doi: 10.3390/genes10110851 (PMC6896034; doi:10.3390/genes10110851)
Supplement: Supplementary file 1 [file genes-10-00851-s001.zip › Table S5. Upper and lower 95% confidence intervals calculated by bootstrapping for Fis values.pdf]

Table S5. Upper and lower 95% confidence intervals calculated by bootstrapping for F_{IS} values for Barrow Island (BWI), Dryandra (DRY) and the Matuwa translocated population of *Bettongia lesueur* between 2011 and 2018.

	F_{IS}	Lower Cl	Upper Cl
BWI	0.050	0.045	0.056
DRY	0.012	0.006	0.018
2011	0.250	0.244	0.256
2012	0.072	0.068	0.077
2013	0.121	0.117	0.125
2014	0.049	0.045	0.053
2015	0.019	0.014	0.024
2016	0.037	0.032	0.042
2017	0.040	0.034	0.045
2018	0.042	0.037	0.047
