# Supplementary material for: Population Genomics of Bettongia lesueur: Admixing Increases Genetic Diversity with no Evidence of Outbreeding Depression
Source: Genes (Basel). 2019 Oct 28;10(11):851. doi: 10.3390/genes10110851 (PMC6896034; doi:10.3390/genes10110851)
Supplement: Supplementary file 1 [file genes-10-00851-s001.zip › Table S6. Estimated membership to a particular cluster assuming two admixed populations.pdf]

Table S6. Estimated membership to a particular cluster assuming two admixed populations for each individual from the founder populations (BWI and DRY) and the translocated population from 2010 to 2018 estimated using fastSTRUCTURE and gghybrid. Hybrid class determined using NEWHYBRIDS is also shown for the translocated population: pure-bred Barrow Island (BWI), pure-bred Dryandra (DRY), backcross to Barrow Island (F_1×BWI), F_1 hybrid (F_1), F_2 hybrid (F_2), backcross to Dryandra (F_1×DRY) and pure-bred Dryandra (DRY).

Population	Individual ID	fastSTRUCTURE		gghybrid		Hybrid Class
		BWI	DRY	BWI	DRY	
**Founder population**						
**BWI**	D5991	0.99920	0.00080	0.99994	0.00006	BWI founder
	D5992	0.99930	0.00070	0.99995	0.00005	BWI founder
	D5993	0.99900	0.00100	0.99994	0.00006	BWI founder
	D5996	0.99920	0.00080	0.99995	0.00005	BWI founder
	D5997	0.99710	0.00290	0.99285	0.00715	BWI founder
	D5998	0.99920	0.00080	0.99995	0.00005	BWI founder
	D6000	0.99920	0.00080	0.99995	0.00005	BWI founder
	D6001	0.99930	0.00070	0.99995	0.00005	BWI founder
	D6003	0.99930	0.00070	0.99995	0.00005	BWI founder
	D6004	0.99880	0.00120	0.99992	0.00008	BWI founder
	D6005	0.99900	0.00100	0.99994	0.00006	BWI founder
	D6006	0.99930	0.00070	0.99995	0.00005	BWI founder
	D6007	0.99930	0.00070	0.99995	0.00005	BWI founder
	D6010	0.99940	0.00060	0.99995	0.00005	BWI founder
	D6011	0.99850	0.00150	0.99801	0.00200	BWI founder
	D6012	0.99930	0.00070	0.99995	0.00005	BWI founder
	D6015	0.99930	0.00070	0.99995	0.00005	BWI founder
	D6016	0.99920	0.00080	0.99995	0.00005	BWI founder
	D6017	0.99930	0.00070	0.99996	0.00005	BWI founder
	D6018	0.99920	0.00080	0.99995	0.00005	BWI founder
	D6019	0.99890	0.00110	0.99995	0.00005	BWI founder
	D6020	0.99880	0.00120	0.99990	0.00010	BWI founder
**Dryandra**	D6021	0.00000	1.00000	0.00005	0.99995	DRY founder
	D6022	0.00000	1.00000	0.00005	0.99995	DRY founder
	D6023	0.00000	1.00000	0.00004	0.99996	DRY founder
	D6024	0.00000	1.00000	0.00005	0.99996	DRY founder
	D6026	0.00000	1.00000	0.00004	0.99996	DRY founder
	D6027	0.00000	1.00000	0.00005	0.99996	DRY founder
	D6028	0.00000	1.00000	0.00005	0.99995	DRY founder
	D6029	0.00000	1.00000	0.00005	0.99996	DRY founder
	D6031	0.00000	1.00000	0.00005	0.99996	DRY founder
	D6032	0.00000	1.00000	0.00005	0.99996	DRY founder
	D6033	0.00000	1.00000	0.00005	0.99996	DRY founder

	D6036	0.00000	1.00000	0.00005	0.99996	DRY founder
	D6038	0.00000	1.00000	0.00004	0.99996	DRY founder
	D6039	0.00000	1.00000	0.00005	0.99996	DRY founder
	D6040	0.00000	1.00000	0.00005	0.99995	DRY founder
	D6041	0.00000	1.00000	0.00005	0.99995	DRY founder
	D6042	0.00000	1.00000	0.00005	0.99995	DRY founder
	D6043	0.00000	1.00000	0.00005	0.99995	DRY founder
	D6044	0.00000	1.00000	0.00005	0.99996	DRY founder
	D6047	0.00000	1.00000	0.00005	0.99996	DRY founder
	D6048	0.00000	1.00000	0.00005	0.99996	DRY founder
	D6050	0.00000	1.00000	0.00005	0.99995	DRY founder
	D6063	0.00000	1.00000	0.00005	0.99996	DRY founder
	D6064	0.00000	1.00000	0.00005	0.99995	DRY founder
**Translocated population**						
**2010**	D5940	0.99760	0.00240	0.99995	0.00005	BWI
	D5941	0.99500	0.00500	0.99995	0.00005	BWI
	D5942	0.99600	0.00400	0.99995	0.00005	BWI
	D5943	0.99860	0.00140	0.99995	0.00005	BWI
**2011**	D5936	0.00000	1.00000	0.00004	0.99996	DRY
	D5937	0.99940	0.00060	0.99995	0.00005	BWI
	D5938	0.49390	0.50610	0.50575	0.49426	F1
	D5945	0.99400	0.00600	0.99995	0.00005	BWI
	D5946	0.99540	0.00460	0.99995	0.00005	BWI
	D5947	0.99660	0.00340	0.99995	0.00005	BWI
	D5948	0.99640	0.00360	0.99995	0.00005	BWI
	D5949	0.00000	1.00000	0.00005	0.99996	DRY
	D5950	0.48410	0.51590	0.49598	0.50402	F1
	D5951	0.99730	0.00270	0.99995	0.00005	BWI
	D5952	0.99940	0.00060	0.99995	0.00005	BWI
	D5953	0.99760	0.00240	0.99995	0.00005	BWI
	D5954	0.99940	0.00060	0.99995	0.00005	BWI
	D5955	0.99760	0.00240	0.99995	0.00005	BWI
	D5956	0.00250	0.99750	0.00005	0.99996	DRY
**2012**	D5934	0.49040	0.50960	0.50241	0.49759	F1
	D5935	0.99690	0.00310	0.99995	0.00005	BWI
	D5957	0.23910	0.76090	0.24104	0.75896	F_1×DRY
	D5958	0.99820	0.00180	0.99995	0.00005	BWI
	D5959	0.99910	0.00090	0.99995	0.00005	BWI
	D5960	0.30100	0.69900	0.30752	0.69248	F_1×DRY
	D5961	0.00000	1.00000	0.00005	0.99996	DRY
	D5962	0.99740	0.00260	0.99995	0.00005	BWI
	D5963	0.48280	0.51720	0.49385	0.50615	F1
	D5964	0.99840	0.00160	0.99995	0.00005	BWI

	D5965	0.49990	0.50010	0.50897	0.49103	F1
	D5966	0.00200	0.99800	0.00005	0.99995	DRY
	D5967	0.48150	0.51850	0.49222	0.50778	F1
	D5969	0.69440	0.30560	0.71439	0.28561	F1×BWI
	D5970	0.49450	0.50550	0.50550	0.49450	F1
	D5971	0.49030	0.50970	0.49872	0.50128	F1
	D5972	0.48150	0.51850	0.48991	0.51009	F1
	D5973	0.00000	1.00000	0.00004	0.99996	DRY
	D5974	0.19160	0.80840	0.18840	0.81160	F_1×DRY
**2013**	D5877	0.99630	0.00370	0.99995	0.00005	BWI
	D5878	0.99870	0.00130	0.99995	0.00005	BWI
	D5914	0.55360	0.44640	0.57142	0.42858	F2
	D5915	0.49650	0.50350	0.50562	0.49438	F1
	D5916	0.00310	0.99690	0.00005	0.99995	DRY
	D5917	0.99820	0.00180	0.99995	0.00005	BWI
	D5918	0.47940	0.52060	0.48982	0.51018	F1
	D5919	0.23830	0.76170	0.23746	0.76255	F_1×DRY
	D5920	0.48180	0.51820	0.49065	0.50935	F1
	D5922	0.69310	0.30690	0.70878	0.29122	F1×BWI
	D5925	0.45190	0.54810	0.46310	0.53690	F2
	D5927	0.26570	0.73430	0.26646	0.73354	F_1×DRY
	D5929	0.32750	0.67250	0.33097	0.66903	F_1×DRY
	D5930	0.69660	0.30340	0.71251	0.28749	F1×BWI
	D5931	0.64770	0.35230	0.66128	0.33872	F1×BWI
	D5932	0.68980	0.31020	0.70582	0.29418	F1×BWI
	D5975	0.00000	1.00000	0.00005	0.99995	DRY
	D5976	0.00010	0.99990	0.00005	0.99996	DRY
	D5977	0.99710	0.00290	0.99995	0.00005	BWI
	D5978	0.00210	0.99790	0.00005	0.99995	DRY
	D5979	0.99720	0.00280	0.99995	0.00005	BWI
	D5980	0.47980	0.52020	0.49041	0.50959	F1
	D5981	0.00090	0.99910	0.00005	0.99996	DRY
	D5984	0.50050	0.49950	0.50766	0.49235	F1
	D5985	0.60410	0.39590	0.61974	0.38026	F2
	D5987	0.00000	1.00000	0.00005	0.99996	DRY
	D5988	0.00000	1.00000	0.00005	0.99996	DRY
	D5989	0.74390	0.25610	0.76276	0.23724	F1×BWI
	D5990	0.14920	0.85080	0.14449	0.85551	F_1×DRY
	D6051	0.42380	0.57620	0.42922	0.57079	F2
	D6052	0.99800	0.00200	0.99995	0.00005	BWI
	D6053	0.99610	0.00390	0.99995	0.00005	BWI
	D6054	0.48700	0.51300	0.50107	0.49893	F1
	D6055	0.50010	0.49990	0.50796	0.49204	F1

	D6056	0.99830	0.00170	0.99995	0.00005	F1
	D6057	0.99510	0.00490	0.99995	0.00005	F1
	D6058	0.24480	0.75520	0.24431	0.75569	F_1×DRY
**2014**	D5809	0.70200	0.29800	0.71986	0.28015	F1×BWI
	D5810	0.91440	0.08560	0.93211	0.06790	BWI
	D5811	0.99770	0.00230	0.99995	0.00005	BWI
	D5812	0.00040	0.99960	0.00005	0.99995	DRY
	D5813	0.68690	0.31310	0.70384	0.29616	F1×BWI
	D5814	0.31940	0.68060	0.32032	0.67968	F_1×DRY
	D5815	0.25530	0.74470	0.25870	0.74130	F_1×DRY
	D5816	0.80390	0.19610	0.82173	0.17828	F1×BWI
	D5817	0.61370	0.38630	0.62843	0.37157	F2
	D5818	0.79000	0.21000	0.81003	0.18997	F1×BWI
	D5819	0.99830	0.00170	0.99995	0.00005	BWI
	D5820	0.49470	0.50530	0.50649	0.49352	F1
	D5821	0.53390	0.46610	0.54619	0.45381	F2
	D5822	0.99370	0.00630	0.99995	0.00005	BWI
	D5855	0.44370	0.55630	0.45392	0.54608	F2
	D5856	0.99560	0.00440	0.99995	0.00005	BWI
	D5857	0.18700	0.81300	0.18276	0.81724	F_1×DRY
	D5859	0.40770	0.59230	0.41513	0.58487	F1
	D5860	0.49030	0.50970	0.50166	0.49834	F1
	D5861	0.75840	0.24160	0.77562	0.22438	F1×BWI
	D5862	0.28850	0.71150	0.28796	0.71204	F_1×DRY
	D5863	0.99670	0.00330	0.99995	0.00005	BWI
	D5865	0.71750	0.28250	0.73373	0.26627	F1×BWI
	D5866	0.75920	0.24080	0.77937	0.22063	F1×BWI
	D5867	0.75550	0.24450	0.77413	0.22587	F1×BWI
	D5868	0.49370	0.50630	0.50067	0.49933	F1
	D5869	0.49770	0.50230	0.50354	0.49646	F1
	D5871	0.45660	0.54340	0.46665	0.53335	F2
	D5872	0.99600	0.00400	0.99995	0.00005	BWI
	D5874	0.41110	0.58890	0.41887	0.58113	F1
	D5875	0.73170	0.26830	0.74741	0.25259	F1×BWI
	D5876	0.34120	0.65880	0.34374	0.65626	F2
	D5913	0.29890	0.70110	0.30380	0.69620	F_1×DRY
**2015**	D5823	0.47110	0.52890	0.48057	0.51943	F2
	D5824	0.78540	0.21460	0.80419	0.19581	F1×BWI
	D5825	0.99810	0.00190	0.99995	0.00005	BWI
	D5826	0.22980	0.77020	0.23075	0.76926	F_1×DRY
	D5827	0.84420	0.15580	0.86005	0.13996	F1×BWI
	D5828	0.17290	0.82710	0.16924	0.83076	F_1×DRY
	D5829	0.49440	0.50560	0.50425	0.49575	F1

	D5850	0.61660	0.38340	0.63055	0.36945	F1×BWI
	D5851	0.31250	0.68750	0.31773	0.68227	F_1×DRY
	D5852	0.80670	0.19330	0.82549	0.17451	F1×BWI
	D5854	0.50020	0.49980	0.51096	0.48904	F1
	D5912	0.59790	0.40210	0.60957	0.39043	F2
	D6059	0.49360	0.50640	0.50488	0.49512	F1
	D6060	0.31870	0.68130	0.31991	0.68009	F_1×DRY
	D6061	0.99820	0.00180	0.99995	0.00005	BWI
	D6062	0.66110	0.33890	0.67631	0.32369	F1×BWI
**2016**	D5830	0.37750	0.62250	0.38391	0.61609	F2
	D5831	0.66920	0.33080	0.68465	0.31535	F1×BWI
	D5832	0.62950	0.37050	0.64197	0.35804	F1×BWI
	D5843	0.53670	0.46330	0.55006	0.44994	F2
	D5844	0.99700	0.00300	0.99995	0.00005	BWI
	D5845	0.69280	0.30720	0.70863	0.29138	F1×BWI
	D5846	0.31670	0.68330	0.32089	0.67911	F2
	D5847	0.99830	0.00170	0.99995	0.00005	BWI
	D5848	0.77930	0.22070	0.79911	0.20090	F1×BWI
	D5849	0.48800	0.51200	0.49998	0.50002	F1
	D5883	0.57060	0.42940	0.58283	0.41717	F2
	D5884	0.53430	0.46570	0.54665	0.45335	F2
	D5888	0.32140	0.67860	0.32604	0.67396	F_1×DRY
	D5889	0.53420	0.46580	0.54331	0.45669	F2
	D5891	0.56800	0.43200	0.57743	0.42257	F2
**2017**	D5833	0.75030	0.24970	0.76763	0.23237	F1×BWI
	D5834	0.99860	0.00140	0.99995	0.00005	BWI
	D5835	0.84140	0.15860	0.85980	0.14020	F1×BWI
	D5836	0.99780	0.00220	0.99995	0.00005	BWI
	D5837	0.56730	0.43270	0.57820	0.42181	F2
	D5838	0.99930	0.00070	0.99995	0.00005	BWI
	D5839	0.90360	0.09640	0.91939	0.08061	F1×BWI
	D5840	0.75840	0.24160	0.76818	0.23182	F1×BWI
	D5842	0.74900	0.25100	0.76817	0.23184	F1×BWI
	D5882	0.49770	0.50230	0.50990	0.49011	F1
	D5886	0.66910	0.33090	0.68271	0.31729	F1×BWI
	D5887	0.76940	0.23060	0.78296	0.21704	F1×BWI
	D5890	0.64120	0.35880	0.65773	0.34227	F1×BWI
	D5892	0.99690	0.00310	0.99995	0.00005	BWI
	D5909	0.24350	0.75650	0.24098	0.75902	F_1×DRY
**2018**	D5881	0.75370	0.24630	0.76723	0.23277	F1×BWI
	D5893	0.60580	0.39420	0.61709	0.38291	BWI
	D5895	0.39790	0.60210	0.40686	0.59314	F2
	D5896	0.41770	0.58230	0.42415	0.57585	F2

	D5897	0.99680	0.00320	0.99995	0.00005	BWI
	D5898	0.70410	0.29590	0.72210	0.27790	F1×BWI
	D5899	0.40860	0.59140	0.41727	0.58273	F2
	D5900	0.58920	0.41080	0.59932	0.40068	F2
	D5901	0.82460	0.17540	0.84250	0.15750	F1×BWI
	D5902	0.78300	0.21700	0.80223	0.19777	F1×BWI
	D5904	0.49710	0.50290	0.50525	0.49475	F1
	D5905	0.83270	0.16730	0.85455	0.14545	F1×BWI
	D5906	0.99870	0.00130	0.99995	0.00005	BWI
	D5907	0.72770	0.27230	0.74385	0.25615	F1×BWI
	D5908	0.88700	0.11300	0.90183	0.09817	F1×BWI
