# Supplementary material for: Population Genomics of Bettongia lesueur: Admixing Increases Genetic Diversity with no Evidence of Outbreeding Depression
Source: Genes (Basel). 2019 Oct 28;10(11):851. doi: 10.3390/genes10110851 (PMC6896034; doi:10.3390/genes10110851)
Supplement: Supplementary file 1 [file genes-10-00851-s001.zip › Table S7. Apparent survival and recruitment rate estimates and 95% confidence intervals.pdf]

Table S7. Apparent survival and recruitment rate estimates ± standard error for founding individuals of *Bettongia lesueur* from each founder population and each hybrid class of the translocated population: pure-bred Barrow Island (BWI), pure-bred Dryandra (DRY), backcross to Barrow Island (F_1×BWI), F_1 hybrid (F_1), F_2 hybrid (F_2), backcrossed to Dryandra (F_1×DRY) and pure-bred Dryandra (DRY). 95% confidence intervals are in parentheses.

	Apparent survival		Recruitment rate
	Female	Male	
**Founder populations**			
BWI	0.937±0.022 (0.855, 0.960)	0.928±0.025 (0.836, 0.954)	0.201±0.046 (0.157, 0.380)
DRY	0.916±0.029 (0.814, 0.945)	0.904±0.032 (0.790, 0.937)	0.050±0.012 (0.039, 0.096)
**Matuwa population**			
BWI	0.944±0.021 (0.867, 0.965)	0.935±0.023 (0.849, 0.959)	0.133±0.033 (0.099, 0.260)
F_1×BWI	0.960±0.016 (0.898, 0.976)	0.953±0.019 (0.883, 0.972)	0.200±0.049 (0.152, 0.388)
F_1	0.983±0.008 (0.951, 0.991)	0.980±0.009 (0.944, 0.989)	0.134±0.036 (0.098, 0.270)
F_2	0.966±0.015 (0.909, 0.982)	0.961±0.017 (0.893, 0.979)	0.221±0.056 (0.165, 0.435)
F_1×DRY	0.967±0.016 (0.907, 0.983)	0.961±0.018 (0.893, 0.980	0.001±0.00003 (0.000, 0.002)
DRY	0.928±0.030 (0.822, 0.959)	0.918±0.034 (0.799, 0.953)	0.113±0.037 (0.074, 0.249)
